# 

**Published:** 1884-10

**Authors:** 


					﻿Vol. IV.
OCTOBER, 1884.
NO. 10.
THE
flOMCEDPATHlC pHY^lClAfl,
A MONTHLY JOURNAL OF MEDICAL SCIENCE.
“Seek the Truth: come whence it may, cost what it will''
HJ. CT. LIE HL HUE. ID.,
EDITOR.
CONTENTS:
(See inside of Cover.)
PHILADELPHIA:
No’. 129 SOTJCTJEI TELIRTJEEN’TIH: STRTD1DT.
ustzenv ■sroie.ic:
A. L. CHATTERTON, PUBLISHING COMPANY, PUBLISHER’S AGENTS.
LONDON:
ALFRED HEATH & CO., Sole Agents FOR Great Britain,
114 Ebury Street, Eaton Square.
Yearly Subscription, $2.50, invariably in advance.
Entered at the Philadelphia Post-Office as Second* Class Mall Matter.
				

